# The analysis of calcitonin gene-related peptide – a narrow path between useful and misleading findings

**DOI:** 10.1177/0333102420941114

**Published:** 2020-07-20

**Authors:** Jan Hoffmann

**Affiliations:** Basic and Clinical Neuroscience, Institute of Psychiatry, Psychology and Neuroscience, King's College London, London, UK

More than 30 years ago Goadsby and Edvinsson revealed in their landmark experiments that calcitonin gene-related peptide (CGRP) plays an essential role in trigeminal communication (1). In the following years, research revealed that CGRP is not only released upon trigeminal activation but can also activate trigeminal neurons (2). In line with these findings, clinical studies showed that CGRP is released in spontaneous migraine (3,4) and cluster headache (5) attacks and that both headache syndromes can be triggered by infusion of CGRP (6,7) and treated by targeting the CGRP pathway (8–10).

These discoveries have triggered a discussion on whether CGRP may act as a biomarker in primary headache disorders. If that would be the case, one may speculate that CGRP concentrations could potentially reflect disease activity, help differentiating between different types of headache disorders and predict treatment response. In particular, the latter aspect would be very useful as current preventive treatments are only effective in a subgroup of patients and at the moment there is no way to predict for an individual patient the efficacy of a preventive treatment. The experience with CGRP receptor antagonists (gepants) and monoclonal antibodies targeting CGRP or its receptor have fueled this discussion. Several clinical trials with these monoclonal antibodies have clearly shown that while roughly half of the patients experience a 50% reduction in their headache/migraine frequency, in a small subgroup of patients these medications are highly effective, reducing headache/migraine frequency by 75–100%, whereas another subgroup did not experience any improvement at all (9). These findings suggest that CGRP may serve as a biomarker that could predict efficacy. From a pathophysiological perspective, they also suggest that in a subgroup of migraineurs other neuropeptides may be more relevant than CGRP.

Unfortunately, things are not as easy as they may seem and CGRP plasma concentrations are unlikely to ever serve as a relevant biomarker in migraine or cluster headache for many reasons. First, CGRP is quickly degraded by proteases and therefore only has a half-life of a few minutes. It is therefore essential that, upon collection, blood samples are immediately mixed with protease inhibitors such as aprotinin to interrupt this process. Secondly, concentrations of CGRP are very low, ranging close to the detection limits of most commercially available enzyme-linked immunoassays (ELISA) and radioimmunoessays (RIA), which affects their accuracy. In addition, the small amounts released by trigeminal neurons are quickly diluted to a concentration that is difficult to detect if blood is collected from a peripheral vein. To obtain accurate results, blood collection should therefore be performed from the jugular vein. Thirdly, CGRP concentrations vary substantially within one individual as well as between individuals. These variations may be higher than the difference between an activated and a baseline state of trigeminal activity. Fourthly, CGRP is not only relevant in trigeminal neurotransmission and can therefore be released in several clinical conditions. It is also released in several primary headaches (e.g. migraine and cluster headache), while it has not been tested for many other headache syndromes, thereby not being particularly useful when intending to use it to aid differential diagnosis. Fifthly, the increase of CGRP plasma concentration can be cause or consequence of trigeminal activation. Finally, CGRP does not cross the blood-brain barrier. As a result, whatever CGRP concentration is measured in peripheral blood is unlikely to reflect the effect of central actions of CGRP.

Given these difficulties, it becomes clear that while some conclusions can be drawn if sample collection and processing is adequately managed and when pooling the findings of a number of patients, it is virtually impossible to use plasma concentrations of CGRP as a biomarker to assess disease activity or predict treatment efficacy in an individual patient.

In contrast, for research purposes, the understanding of the molecular CGRP pathways, detailed knowledge on the relevant sites of action and the influence of CGRP-dependent mechanisms on trigeminal nociceptive neuronal transmission and on central pain processing is essential to further understand the pathophysiology of headache disorders. Nevertheless, beyond its role in migraine and cluster headache, information on the pathophysiological role of CGRP in other primary as well as in secondary headaches remains scarce.

In their article published in this edition of *Cephalalgia*, Ashina et al. aimed at elucidating the role of CGRP in persistent post-traumatic headache (PTH) attributed to traumatic brain injury (TBI). Since PTH involves trigeminal activation, and given that its clinical presentation frequently has a migrainous phenotype, it appears conceivable that PTH is associated with elevated CGRP levels in plasma. In this context, preclinical studies show that mechanisms involving CGRP drive TBI-related development of central sensitization as well as the response to bright light stress and may therefore be relevant for the expression of PTH (11). In line with these findings, erenumab, a monoclonal antibody targeting the CGRP receptor, has been shown to reduce headache intensity in PTH (12). These findings strongly support a role of CGRP in PTH.

Interestingly, Ashina et al. did not identify an increase of plasma CGRP in patients with PTH (13). This surprising result is difficult to explain in the context of the current understanding of PTH pathophysiology as well as the increasing preclinical and clinical evidence suggesting the opposite. However, several mechanisms may explain these findings. First, it is conceivable that CGRP-dependent mechanisms in PTH rely on an upregulation of CGRP receptor expression or enhancement of another downstream mechanism rather than on the increase of CGRP release. Secondly, preclinical data suggest that CGRP is particularly relevant in the early stages after TBI, increasing vulnerability to develop persisting PTH (11); however, the patients investigated by Ashina et al. had a mean disease duration of 49.3 months. Thirdly, it may be possible that central CGRP receptors may be essential for development of PTH; however, central CGRP release can’t be measured in peripheral blood as it does not cross the blood-brain barrier. Finally, it is conceivable that methodological issues such as, for example, the lack of protease inhibitors and a resulting degradation of CGRP, may explain these findings. On the other hand, the CGRP plasma concentrations observed in this study were relatively high in the healthy volunteer group, suggesting that the RIA may have had a specificity issue. Given these methodological aspects, further studies are needed to confirm these findings and clarify further the role of CGRP in the development of PTH after TBI.
